# Single-crystalline perovskite wafers with a Cr blocking layer for broad and stable light detection in a harsh environment†

**DOI:** 10.1039/c8ra02709a

**Published:** 2018-04-19

**Authors:** Qian Wang, Dongliang Bai, Zhiwen Jin, Shengzhong (Frank) Liu

**Affiliations:** Key Laboratory of Applied Surface and Colloid Chemistry, Ministry of Education, Shaanxi Key Laboratory for Advanced Energy Devices, Shaanxi Engineering Lab for Advanced Energy Technology, School of Materials Science & Engineering, Shaanxi Normal University Xi'an 710119 P. R. China jinzhiwen@snnu.edu.cn; Dalian National Laboratory for Clean Energy, iChEM, Dalian Institute of Chemical Physics, Chinese Academy of Sciences Dalian 116023 P. R. China szliu@dicp.ac.cn

## Abstract

Herein, ultrathin (∼35 μm) CH_3_NH_3_PbI_3_ (MAPbI_3_) single-crystalline wafers have been successfully prepared by using an appropriate geometry-regulated dynamic-flow reaction system. The measurement results proved that the obtained wafers have high crystallinity, and showed broad light absorption from ultraviolet to near infrared (850 nm) which can be attributed to the indirect bandgap. Straight after, such an MAPbI_3_ wafer was used to fabricate high-quality photodetectors (PDs). On account of its faster carrier transport and significantly reduced defect density, the device exhibits a high photoresponse (*R*) of 5 A/W and short on/off response (0.039 s/0.017 s). Interestingly, by introducing a Cr interlayer between the MAPbI_3_ wafer and the Au electrode to avoid the migration of Au, the PD shows nearly no degradation when it works at 200 °C. Furthermore, the device performance shows very little degradation over the course of 60 days of storage under ambient conditions owing to its lack of grain boundaries. We believe the strategy reported here is very promising for achieving broad photodetection in a harsh environment.

## Abstract 2

Perovskite single-crystalline wafer based photodetectors are prepared with broad photodetection, and show little degradation at high-temperature or with storage under ambient-conditions.

## Graphical abstract

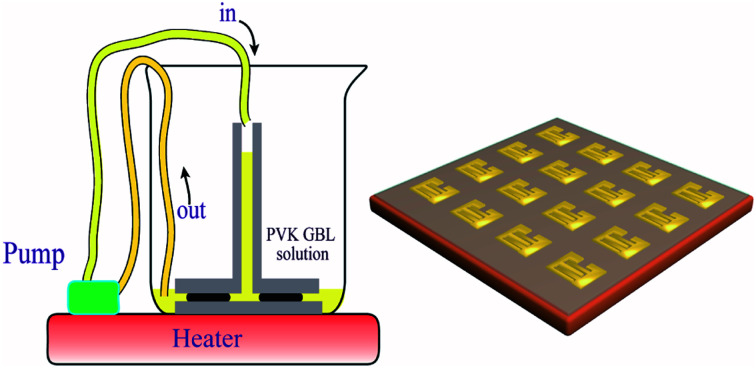

## Introduction

In recent years, halide perovskites (PVK) have attracted much attention due to their merits of high absorption coefficients, long carrier lifetimes, high electron and hole mobility, and low temperature solution processability, *etc.*^1–8^ These merits make the hybrid perovskite a strong competitor in the development of next-generation optoelectronic devices.^9–14^ Compared with the polycrystalline perovskites, the single-crystal perovskites show further enhanced optoelectronic properties due to their having a more long-range and ordered structure.^15–19^

First of all, the redshifted absorption edge is observed due to its indirect-bandgap absorption transition with a bandgap of 60 meV smaller than the direct bandgap:^20–22^ the absorption coefficient corresponding to the below-bandgap transition is several orders of magnitude smaller than that of the above-gap transition.^23^ Hence, a thick perovskite single crystal could absorb more light through below-bandgap.^24^ Meanwhile, the single crystal perovskite show longer lifetime and much longer carrier diffusion length well above tens of micrometer due to the absence of grain boundaries and significantly reduced defect density.^25,26^ Moreover, the carrier mobility in single crystal is increased to 164 cm^2^ V^−1^ s^−1^.^27^ Therefore, the single crystal perovskite can provide not only a wider absorption spectrum but also better carrier transport efficiency, demonstrating its potential in applications as broad photodetector (PD).

In reality, the single crystal perovskite based PD indeed shows better photoelectronic performance than that of the PD made of polycrystalline perovskites,^28^ however, it is still not reach the commercial requirement. As is well known, the polycrystalline perovskites are stable at temperature not exceeding 85 °C.^29^ Meanwhile, the humidity and illumination also proved the main degradation trigger.^30,31^ For the single crystal perovskite, its stability is effectively enhanced under above-mentioned stress conditions for the lack of grain boundaries.^32,33^ However, in addition to the degradation of the perovskite itself, the device architecture is also found to greatly determine the stability of perovskite devices. It is reported that the considerable amounts of Au diffuse from the electrode to the perovskite layer, resulting in the irreversible performance loss.^34,35^ Hence, the issue whether the poor thermal stability of the single crystal perovskite PDs is caused by the diffusion of Au electrode should pay more attention.

In this study, we synthesized the ultrathin (∼35 μm) CH_3_NH_3_PbI_3_ (MAPbI_3_) singly-crystalline wafer, fabricated the corresponding PDs and investigated its stability. Of course, the device exhibits excellent performance with broad photodetection and outstanding ambient stability. Interestingly, the PD with ultrathin Cr interlayer between the wafer and Au electrode presents the excellent thermal stability without any obvious degradation. We believe this finding provides a new key variable component and paves the way toward using perovskite crystals in highly efficient photoelectric device.

## Experimental section

### MAPbI_3_ wafer preparation

Based on the inverse temperature crystallization of perovskites,^36–38^ PbI_2_ and MAI was dissolved in γ-butyrolactone (GBL) with solution concentration controlled at 1.1 M. The reaction system was placed in a flat container. Two thin glass slides were separated and aligned in parallel by two spacers with predefined separation to confine the crystal growth within the slit channel. The single-crystalline wafer thickness is defined by the spacers. When the MAPbI_3_ wafer is taken out from the growth solution, dipped into anhydrous diethylether to dissolve the solvent residue, and finally dried at 60 °C in a vacuum oven. Fig. S1† shows the photograph of one as-grown single crystals perovskite wafer.

### Device Fabrication

PDs were fabricated by directly thermal evaporation of 2 nm chromium and 60 nm-thick gold electrodes through a shadow mask onto the above obtained single crystal wafer which results in a channel width of 4000 μm and a channel lengths of 40 μm.

### Characterization

The morphology and cross-sectional image of the formed wafer were characterized by SEM (Hitachi S-4800). UV-Vis spectra were recorded using JASCO V-570 spectrophotometer. The phase identification was determined by using a Rifaku D/MAX-2004 XRD with Cu Kα radiation (*λ* = 1.54178 Å) operating at 40 KV and 60 mA. All electrical characterizations were recorded with a Keithley 4200 at room temperature in air. Prior to the use of monochromatic light, the spectral response of the mono-silicon solar cell was measured and normalized to the National Renewable Energy Laboratory (NREL) standards.

## Results and discussion

**Fig. 1:**
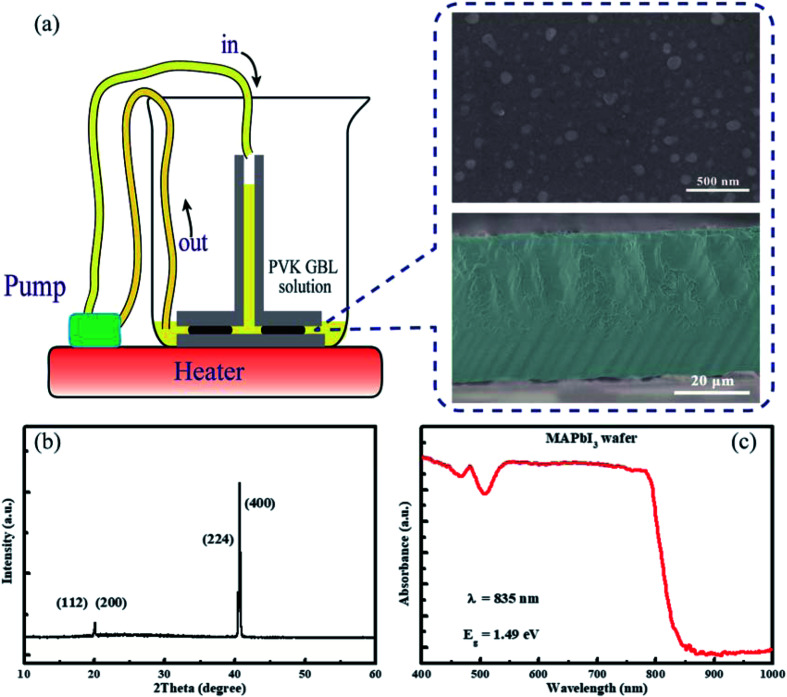
(a) The schematic illustration for preparing the single-crystalline MAPbI_3_ wafer with the inset showing corresponding surface and cross-section SEM images. (b) The XRD pattern and (c) absorption spectrum of MAPbI_3_ wafer.

The ultrathin geometry-defined dynamic-flow reaction system was employed to synthesis single-crystalline MAPbI_3_ wafers,^28^ as shown in Fig. 1a. Usually, the lateral crystal growth can be limited by the inefficient long-range transportation of precursor ions along micrometer-size gap to continuously replenish the depleted precursors by crystal growth.^20^ Here, the dynamic flow is achieved using a peristaltic pump to warrant the crystal growth by enhanced the ion diffusion using constant fresh solution. Finally, the prepared MAPbI_3_ wafers, which were sandwiched by two paralleled glass slides, grow along with the slit channel easily. The thickness of the perovskite wafer was determined by the spacers between the two glass slides and wafer shape was defined by the design of slit channel. And the reaction process was finished in a heated beaker.

The inset of Fig. 1a and S2† give the surface and cross-section scanning electron microscopy (SEM) images of the fabricated MAPbI_3_ wafer. The surface morphology of the single-crystalline MAPbI_3_ wafer shows some nano-grain boundaries as reported by Mohammed *et al.*,^39^ which is very possibly due to the surface hydration and disorder facilitating the ion migration in MAPbI_3_ wafer from bulk to surface.^40^ The cross-section SEM images reveal the thickness of MAPbI_3_ wafer is about ∼35 μm. To detect the structural information and crystalline quality of MAPbI_3_ wafer, the X-ray diffraction (XRD) was employed. The XRD pattern of the synthesized single-crystalline wafer is shown in Fig. 1b, which provide evidence the as-grown MAPbI_3_ wafer was tetrahedral phase, and shown the (112) facets.^41,42^ By scanning (112) facets, diffractions corresponded to {112} crystal planes appearance. It should be mentioned that the synchronized appearance of {112} crystal planes was attributed to their adjacent 2*θ* value (the difference is ∼0.1°). Fig. 1c and S3† provide the absorption spectrum of the single crystal MAPbI_3_ wafer and the polycrystalline film, respectively. The absorption edge is at 850 nm for the single crystal wafer, showing the redshift compared with the polycrystalline film. This result demonstrates the single-crystalline MAPbI_3_ wafer possesses a narrower bandgap, which is in agreement with the report as told above.

**Fig. 2:**
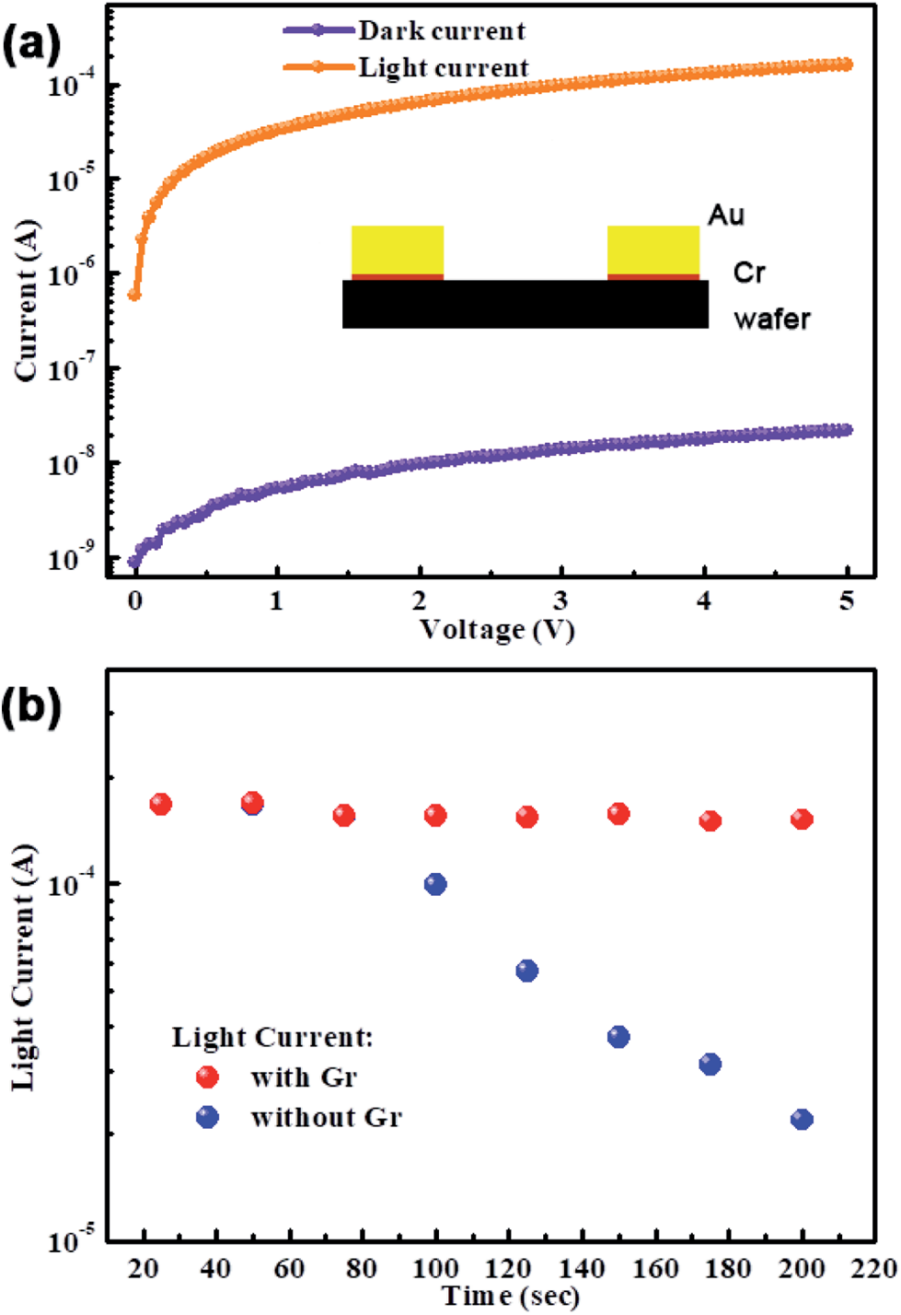
(a) *I*–*V* curves of the PD made of MAPbI_3_ wafer with Cr metal as the interlayer between MAPbI_3_ wafer and Au electrode. (b) The temperature dependent light current stabilities of PDs with and without interlayer Cr (under illumination 20 mW cm^−2^ using an LED laser emitting at 515 nm).

Followed, the MAPbI_3_ wafer was utilized to fabricate the PDs on account of the superior crystallization performance and optical properties. We have designed the PD structure, as shown in the inset of Fig. 2a: MAPbI_3_ wafer was regarded as the active layer, straight after, interdigitated ultra thin 2 nm Cr interlayer and 60 nm Au was directly thermal evaporation as electrode. The current–voltage (*I*–*V*) curves are measured at dark and under illumination 20 mW cm^−2^ using an LED laser emitting at 515 nm (shown in Fig. 2a). At 5 V, the dark current is 2.2 × 10^−8^ A, the larger dark current may caused by the grain boundaries in the surface and the *in situ* ion migration in the wafer as reported.^43^ While under light illumination, the device shows the photocurrent of 1.6 × 10^−4^ A. Obviously, the PD exhibits a high signal-to-noise ratio of about 10^4^. Furthermore, one important parameter, responsivity (*R*), represents the ratio of the photocurrent to the incident light power, is defined to characterize the sensitivity of a PD.^44,45^ According to the following equation:^46,47^1
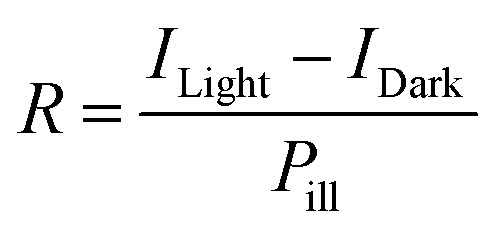
where *I*_Light_ is current under light illumination, *I*_Dark_ the dark current, *P*_ill_ the incident illumination power on the effective area (channel area), the best *R* of 5 A/W is achieved upon device optimization. The results suggest that the MAPbI_3_ wafer based PD is a highly sensitive photoelectric device compared with other perovskite thin film based PDs with such lateral device structure.^48,49^

Fig. 2b compares the temperature stability of the devices with and without Cr interlayer. For the reference device, the photocurrent reduces the one order of magnitude when the temperature increased to 200 °C, which has been proved by the Au migration into the MAPbI_3_ at high temperature.^34,50^ Mostly, the deep trap states were produced by Au atoms within the perovskite semiconductor, which is beneficial for the nonradiative recombination and consequently degrades the photocurrent. However, for the Cr metal induced device, the photocurrent almost keeps constant with elevating temperature. The main reason is that Cr interlayer stop the Au metal diffusion preventing the reduced photoelectric performance.

**Fig. 3:**
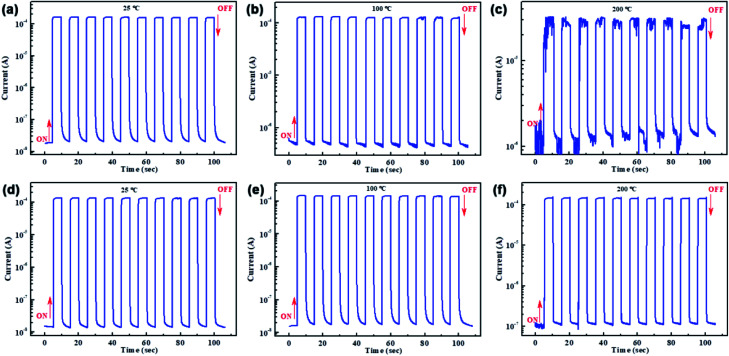
On/off switching properties at different temperature to compare the thermal stability of PDs: (a–c) without and (d–f) with Cr interlayer (in dark condition and under illumination 20 mW cm^−2^ using an LED laser emitting at 515 nm).

The time-dependent photoresponse of the above two PDs measured at different temperature in dark and under illumination 20 mW cm^−2^ using an LED laser emitting at 515 nm are depicted in Fig. 3. The results are in good agreement with Fig. 2b. At room temperature, the photocurrent of the reference PD rapidly increases to a peak value after turning on the light, and then drop to the initial when the light was turned off. The stable periodic response shows that the MAPbI_3_ wafer based PD has highly reproducible characteristics. When increase the temperature to 100 °C, it is obvious that the light current decrease but dark current increase, leading to the lower on/off ratio. More seriously, the high temperature of 200 °C deteriorates the current–time (*I*–*t*) curve, as shown in Fig. 3c, lowering the response of reference PD. This result indicates the poor thermal stability of PD without Cr interlayer, which should be caused by the above told facilitated Au diffusion facilitated at high temperature. However, the PD with Cr interlayer maintains high photoelectric responsiveness along with increasing the temperature, which shows an excellent thermal stability. Therefore, the Au diffusion from electrode to perovskite layer is the main reason for reducing the photoelectric performance of the PD. And the Cr interlayer indeed plays an important role in preventing Au diffusion and avoiding the degradation of photoelectric performance at high temperature.

**Fig. 4:**
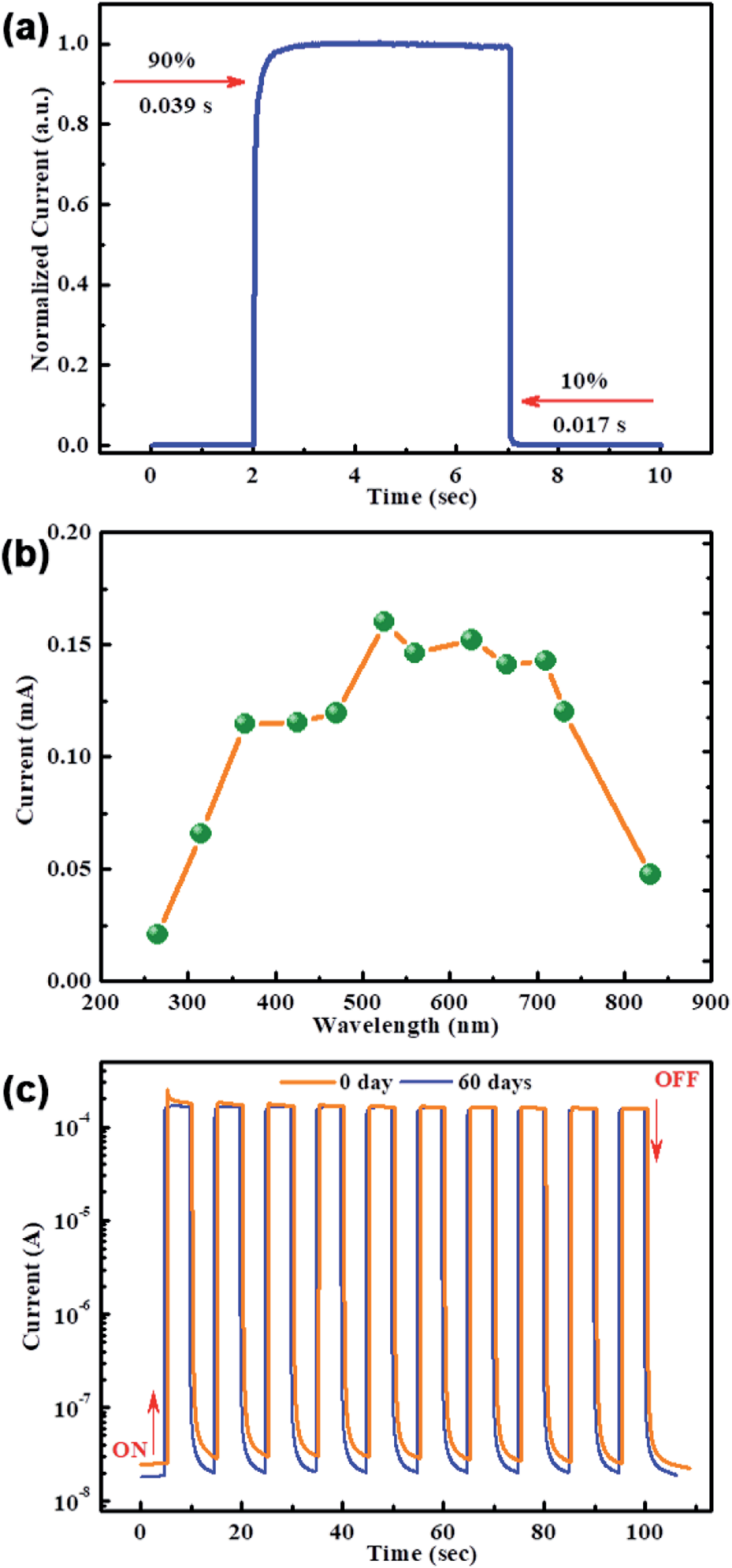
(a) Time-resolved photocurrent and (b) photocurrent spectrum of MAPbI_3_ wafer with Cr interlayer. (c) *I*–*t* curves for the initial and the PD stored in ambient condition after two months.

A cycle of switching photocurrent curve was exhibited in Fig. 4a to investigate the response time of the Cr induced MAPbI_3_ wafer based PD with Cr interlayer. It is well known that the rise time and decay time are respectively defined as the time consumed when the current rise or fall to the 90% of the peak value.^51–53^ The rise time and decay time extracted from Fig. 4a are 0.039 s and 0.017 s, respectively, showing the fast response for the Cr induced MAPbI_3_ wafer the PD with Cr interlayer. The spectral selectivity of the PD is presented in Fig. 4b, which is consistent with its absorption spectrum and shows broad photodetection (UV to 850 nm).^54,55^ Furthermore, the stability of the PD with Cr interlayer is measured to test the practical application. Fig. 4c compares the *I*–*t* curves of the initial device and stored in ambient environment after two months at room temperature. Amusingly, the results demonstrate the excellent stability of the PD with Cr interlayer.

## Conclusions

In summary, we synthesized thin single-crystalline MAPbI_3_ wafer with extended absorption, and fabricated the corresponding PDs. It should be noted that an ultrathin Cr interlayer was induced sandwiched between the wafer and Au electrode to hinder the diffusion of Au electrode under high temperature. As a result, the fabricated device shows high photoresponse (5 A/W), short on/off response time (0.039 s/0.017 s) and broad photodetection region (UV to 850 nm). Moreover, the PD shows nearly no degradation when it works at 200 °C or storage under ambient conditions more than 60 days. This study demonstrates the potential for the application of the perovskite single-crystalline wafer in PDs.

## Author contributions statement

Q. W. and D. B. contributed equally to this work. Q. W. and D. B. performed the experiments, data analysis, and experimental planning. The project was conceived, planned, and supervised by Z. J. and S. L. The manuscript was written by Q. W., Z. J. and S. L. All authors reviewed the manuscript.

## Conflicts of interest

The authors declare no competing financial interests.

## Supplementary Material

RA-008-C8RA02709A-s001

## References

[cit1] Liu Y. (2015). et al.. Adv. Mater..

[cit2] Fang Y. (2015). et al.. Nat. Photonics.

[cit3] Wang Q. (2018). et al.. Adv. Energy Mater..

[cit4] G. X. (2013). et al.. Science.

[cit5] Han Q. (2016). et al.. Adv. Mater..

[cit6] Zhang X. (2018). et al.. ACS Appl. Mater. Interfaces.

[cit7] Bai D. (2018). et al.. ACS Energy Lett..

[cit8] Wang Q. (2017). et al.. ACS Energy Lett..

[cit9] Rao H. S. (2017). et al.. Adv. Mater..

[cit10] Liu Y. (2016). et al.. Adv. Opt. Mater..

[cit11] Chen Y. X. (2016). et al.. J. Am. Ceram. Soc..

[cit12] Elbaz G. A. (2017). et al.. Nano Lett..

[cit13] Jiang J. (2017). et al.. J. Mater. Chem. A.

[cit14] Feng J. (2017). et al.. Nano Energy.

[cit15] Barker A. J. (2017). et al.. ACS Energy Lett..

[cit16] Yang B. (2017). et al.. Adv. Funct. Mater..

[cit17] Dong R. (2015). et al.. Adv. Mater..

[cit18] Wang Q. (2016). et al.. Adv. Mater..

[cit19] Chen M. (2017). et al.. ACS Nano.

[cit20] Chen Z. (2017). et al.. Nat. Commun..

[cit21] Wang T. (2017). et al.. Energy Environ. Sci..

[cit22] Hutter E. M. (2016). et al.. Nat. Mater..

[cit23] Motta C. (2015). et al.. Nat. Commun..

[cit24] Saidaminov M. I. (2016). et al.. Adv. Mater..

[cit25] Shi D. (2015). et al.. Science.

[cit26] Saidaminov M. I. (2015). et al.. Nat. Commun..

[cit27] Dong Q. (2015). et al.. Science.

[cit28] Liu Y. (2016). et al.. Adv. Mater..

[cit29] Li X. (2015). et al.. Nat. Chem..

[cit30] Zhang J. (2018). et al.. Adv. Energy Mater..

[cit31] Jiang J. (2018). et al.. Adv. Energy Mater..

[cit32] Liu Y. (2018). et al.. Adv. Sci..

[cit33] Murali B. (2017). et al.. J. Phys. Chem. Lett..

[cit34] Domanski K. (2016). et al.. ACS Nano.

[cit35] Hu X. (2017). et al.. Nanoscale.

[cit36] Kadro J. M. (2015). et al.. Sci. Rep..

[cit37] Zhang T. (2015). et al.. Chem. Commun..

[cit38] Saidaminov M. I. (2015). et al.. Chem. Commun..

[cit39] Sarmah S. P. (2017). et al.. Nano Lett..

[cit40] Murali B. (2016). et al.. ACS Energy Lett..

[cit41] Zuo Z. (2018). et al.. Mater. Res. Bull..

[cit42] De A. (2017). et al.. Nanoscale.

[cit43] Geske T. (2017). et al.. Adv. Funct. Mater..

[cit44] Jin Z. (2016). et al.. Adv. Mater..

[cit45] Jin Z. (2016). et al.. ACS Appl. Mater. Interfaces.

[cit46] Zhang X. (2017). et al.. Nanoscale.

[cit47] Pang L. (2018). et al.. Part. Part. Syst. Charact..

[cit48] Leung S. F. (2018). et al.. Adv. Mater..

[cit49] Lafalce E. (2016). et al.. ACS Appl. Mater. Interfaces.

[cit50] Cacovich S. (2017). et al.. Nanoscale.

[cit51] Jin Z. (2014). et al.. Sci. Rep..

[cit52] Zhang J. (2017). et al.. RSC Adv..

[cit53] Gao Z. (2018). et al.. Carbon.

[cit54] Jin Z., Wang J. (2013). J. Mater. Chem. C.

[cit55] Wang Q. (2017). et al.. Adv. Mater. Technol..

