# Supplementary material for: Single-crystalline perovskite wafers with a Cr blocking layer for broad and stable light detection in a harsh environment
Source: RSC Adv. 2018 Apr 19;8(27):14848–53. doi: 10.1039/c8ra02709a (PMC9079962; doi:10.1039/c8ra02709a)
Supplement: RA-008-C8RA02709A-s001 [file RA-008-C8RA02709A-s001.pdf]

Electronic Supplementary Material (ESI) for RSC Advances.
This journal is © The Royal Society of Chemistry 2018

Supporting Information

Single-Crystalline Perovskite Wafers with Cr blocking layer for Broad and Stable Light Detection in Harsh Environment

Qian Wang[1,#], Dongliang Bai[1#], Zhiwen Jin[1,*], and Shengzhong (Frank) Liu[1,2,*]

These authors contributed equally to this work.
[1]Key Laboratory of Applied Surface and Colloid Chemistry, Ministry of Education; Shaanxi Key Laboratory for Advanced Energy Devices; Shaanxi Engineering Lab for Advanced Energy Technology; School of Materials Science & Engineering, Shaanxi Normal University, Xi'an, 710119, P. R. China.
E-mail: jinzhiwen@snnu.edu.cn

[2]Dalian National Laboratory for Clean Energy; iChEM, Dalian Institute of Chemical Physics, Chinese Academy of Sciences, Dalian, 116023, P. R. China
E-mail: szliu@dicp.ac.cn

Keywords: single crystal, perovskite, wafer, photodetector, stability

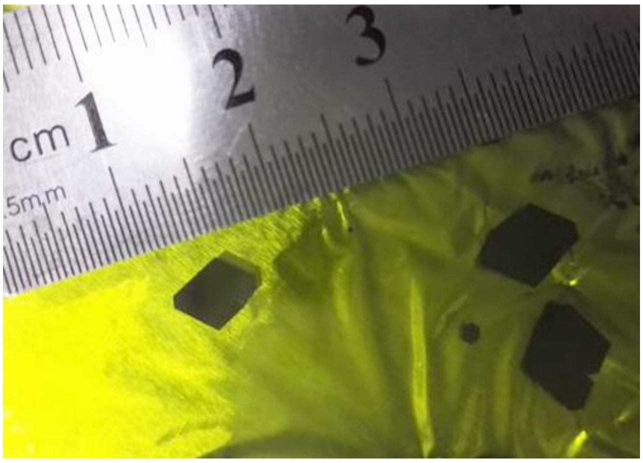

Figure S1. Photograph for the as-grown single crystals perovskite wafer.

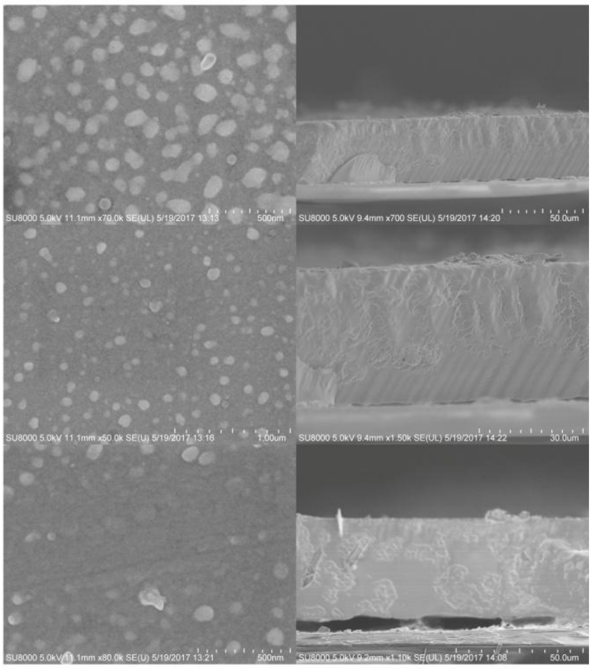

Figure S2. Surface and cross-section SEM images for the wafer at different parts.

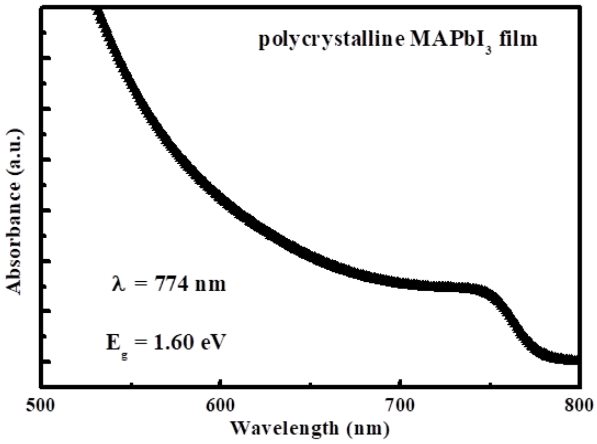

Figure S3. Absorption spectrum of the polycrystalline $MAPbI_3$ film.
